# *KIF1A*-associated neurological disorders: therapeutic opportunities and challenges

**DOI:** 10.1038/s41431-025-01978-8

**Published:** 2025-11-27

**Authors:** Qing Lin, Dylan Verden, John Christodoulou, Wendy A. Gold, Simranpreet Kaur

**Affiliations:** 1 Department of Paediatrics, The University of Melbourne, Melbourne, VIC Australia; 2 Brain and Mitochondrial Research Group, Murdoch Children’s Research Institute, The Royal Children’s Hospital, Melbourne, VIC Australia; 3 KIF1A.ORG, New York, USA; 4 Victorian Clinical Genetics Services, The Royal Children’s Hospital, Melbourne, VIC Australia; 5 Molecular Neurobiology Research Laboratory, Kids Research, The Children’s Hospital at Westmead, and The Children’s Medical Research Institute, Sydney, NSW Australia; 6 Neuroscience theme, School of Medical Sciences, Faculty of Medicine and Health, University of Sydney, Sydney, NSW Australia; 7 Molecular Neurobiology Research Group, Kids Research, The Children’s Hospital at Westmead, The Sydney Children’s Hospital Network, Sydney, NSW Australia

**Keywords:** Translational research, Paediatric neurological disorders

## Abstract

*KIF1A*-Associated Neurological Disorder (KAND) is a rare, progressive neurodegenerative condition caused by variants in the *KIF1A* gene, which encodes a kinesin-3 motor protein essential for anterograde axonal transport of synaptic vesicles, dense core vesicles, and organelles in neurons. KAND comprises a broad spectrum of overlapping neurological phenotypes, including hereditary spastic paraplegia, intellectual disability, peripheral neuropathy, optic nerve atrophy, epilepsy, and progressive motor decline. Pathogenic variants in *KIF1A* disrupt the balance of intracellular transport and neuronal signalling through diverse mechanisms, manifesting with highly variable disease onset, severity, and clinical progression. Although advances in genomic testing have improved diagnosis, reported KAND cases remain concentrated in developed countries, highlighting ongoing global inequities in access to diagnosis and care. At present, no cure exists for KAND; treatment is limited to symptom management. A deeper understanding of KIF1A function, supported by the development of robust cellular and animal models, is critical for therapeutic development. This review summarises the clinical and molecular features of KAND and highlights current and emerging strategies aimed at slowing disease progression or correcting its underlying causes. We emphasise the urgent need for targeted treatment strategies addressing the heterogeneity of KAND.

## Introduction

Kinesins are a family of molecular motors that play crucial roles in intracellular transport by moving cargoes along microtubules. *Kinesin-3 family member 1A (KIF1A)* is specifically expressed in neurons and is responsible for the microtubule-based, fast, anterograde transport over long distances of various payloads, including synaptic vesicles, synaptic vesicle precursors, dense core vesicles (DCVs), lysosomes, and other organelles [1, 2]. This transport is essential for neuronal development, synaptic function, and overall neuronal survival. Pathogenic variants in the *KIF1A* gene disrupt this transport, causing a delay in the delivery of key cargoes, triggering a cascade of adverse neurological effects, ultimately resulting in severe brain disorders collectively referred to as *KIF1A*-Associated Neurological Disorder (KAND).

Initially, disease-causing genetic changes in the *KIF1A* gene were linked to specific neurological disorders, including Hereditary Spastic Paraplegia (HSP/SPG30; MIM 610357 and 620607), Hereditary Sensory and Autonomic Neuropathy (HSAN) IIC (MIM 614213) and Neurodegeneration and Spasticity with or without Cerebellar Atrophy or Cortical Visual impairment (NESCAV; MIM 614255) syndrome. However, advances in genetic testing have expanded the clinical phenotypes associated with *KIF1A* variants to include neurodevelopmental disorders such as Rett Syndrome [3], PEHO syndrome [4] and Charcot-Marie-Tooth disease [5]. Further understanding of the role of KIF1A in neuronal function has revealed that variants in *KIF1A* now contribute to a much broader spectrum of neurodevelopmental and neurodegenerative disorders. This expanded knowledge highlights the diverse and often overlapping clinical manifestations associated with KAND. This growing body of evidence has led to the recognition of KAND as an umbrella term encompassing all neurological conditions caused by *KIF1A* variants. KAND is now understood to present a wide range of overlapping clinical features, including cognitive impairment, motor dysfunction, and neurodegeneration. Recognising KAND as a distinct clinical entity has enhanced our ability to diagnose and manage patients with *KIF1A*-related disorders, emphasising its importance in the differential diagnosis of complex neurological conditions.

## Main body

### *KIF1A*-associated neurological disorder (KAND)

Since 2016, the efforts of the parent-led KIF1A.ORG Foundation [6] and its research network members have facilitated the confirmed genetic diagnosis of more than 550 individuals. However, the clinical overlap of KAND with other neurological disorders increases the risk of patients being diagnosed as having cerebral palsy [7]. Moreover, the diagnostic confirmation of KAND relies exclusively on genetic testing, which is often limited to specialised laboratories. The lack of timely access to advanced genomic sequencing technologies further contributes to the failure to diagnose KAND. This challenge is particularly evident in regions with limited healthcare resources and genetic diagnostic infrastructure. For example, in the largest cohort of KAND patients reported to date (*n* = 177) by Sudnawa et al., 61% of individuals were from North America, 23.5% from Australia, and 10.5% from Europe, whereas only 5% of cases were reported from South America and Asia, and no cases from Africa [8]. Given the population sizes and genetic diversity of these latter regions, this disparity suggests a significant underrepresentation of KAND cases; insufficient access to genetic testing may be a factor, but language barriers and knowledge gaps regarding epidemiological infrastructure in these regions likely also play a role. These findings underscore the need for broader implementation of genomic sequencing and increased awareness of KAND in underserved populations, as the true prevalence of KAND is likely much higher than current estimates.

**Fig. 1:**
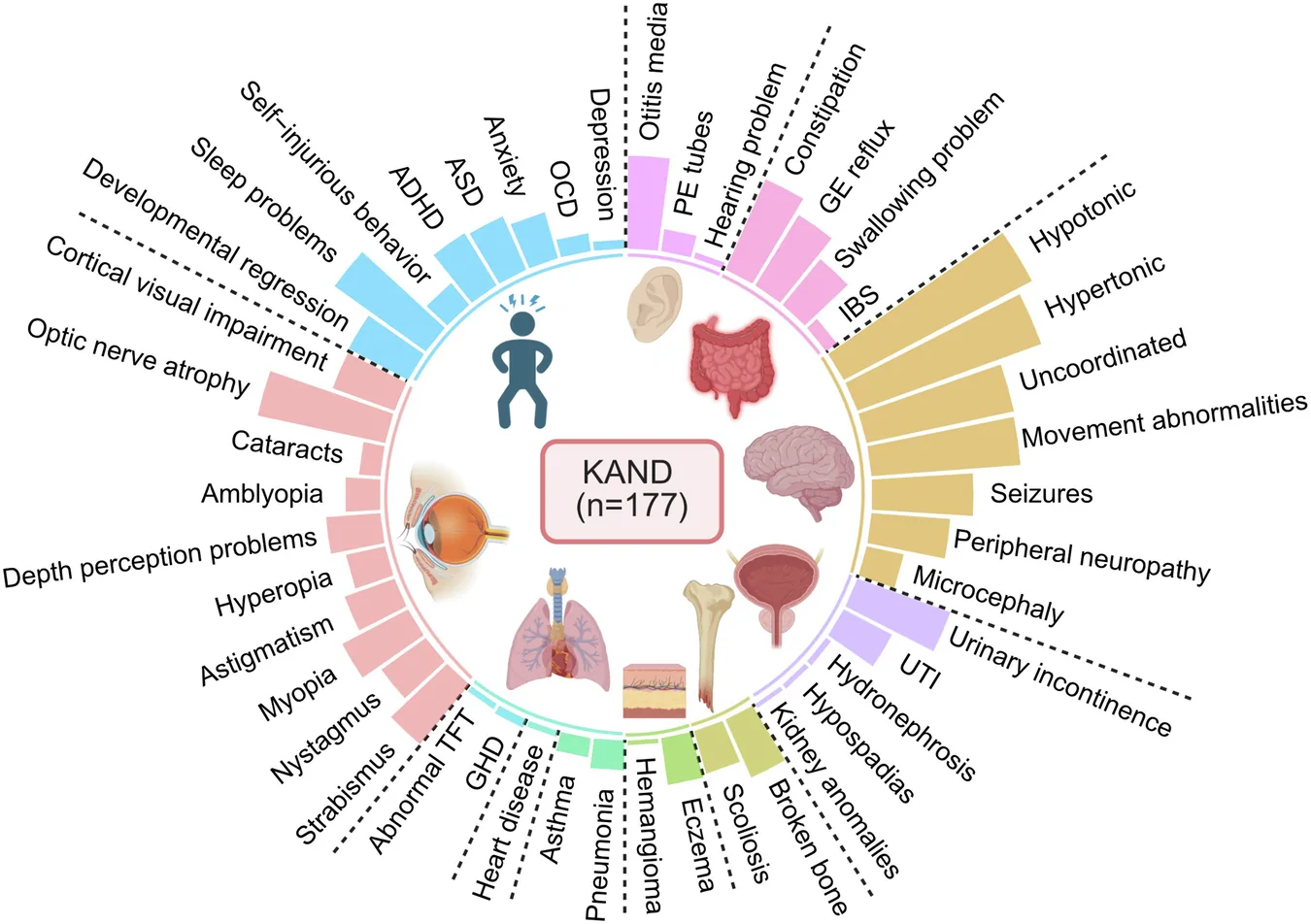
KAND is a multi-system disorder. This figure illustrates the multi-systemic nature of *KIF1A*-Associated Neurological Disorder (KAND). The schematic represents an overall summary of caregiver-reported data from 177 KAND cases [8], highlighting the diverse clinical manifestations of the disorder. Due to the heterogeneity of KAND, not all symptoms are present in every individual. In addition, these data are derived from a cohort of patients with missense variants in the motor domain. Other clinical and genetic entities, such as HSAN II and ALS, are described in the text but not represented in this figure. Illustration created using R Studio and in BioRender. Christodoulou (2025) https://BioRender.com/j55y416. GE gastroesophageal, IBS irritable bowel syndrome, UTI urinary tract infection, GHD growth hormone deficiency, TFT thyroid function test, ADHD attention deficit hyperactivity disorder, ASD autistic spectrum disorders, OCD obsessive–compulsive disorder.

In addition to global developmental delay and severe intellectual disability, individuals affected with KAND exhibit a spectrum of clinical manifestations affecting multiple organ systems, leading to myopathy, peripheral neuropathy, epilepsy, cognitive impairment and brain atrophy, visual problems, gastrointestinal dysfunction and urogenital conditions [8] (Fig. 1).

### Pathophysiology of KAND

#### Gene structure and protein domains

The *KIF1A* gene is located on chromosome 2q37 and is predominantly expressed in the brain and the spinal cord [1]. According to the RNA-sequencing data, the Genotype-Tissue Expression (GTeX) project of human tissues reveals that *KIF1A* is expressed highly in the cerebral cortex, hippocampus, hypothalamus, cerebellum, caudate, and pituitary gland [9]. According to the National Centre for Biotechnology Information, the *KIF1A* gene contains 55 exons, generating up to 33 transcript isoforms (Gene ID: 547, https://www.ncbi.nlm.nih.gov/gene/547). The longest isoform encodes a protein with 1816 amino acid residues (205 kDa, NM_001379631.1, NP_001366560.1), while the shortest encodes 1678 residues (190 kDa, NM_001379641.1, NP_001366570.1). In the GTex portal, the two most expressed isoforms are ENST00000649096.1 (corresponding to NM_004321.8, NP_004312.2, isoform 2) and ENST00000460788.5 (no corresponding transcript and protein in NCBI, contains only 9 exons). The presence of multiple KIF1A isoforms suggests that the protein may have diverse functions or site-specific roles, potentially explaining the broad spectrum of clinical phenotypes associated with *KIF1A* variants. In this study, all variants described use the disease-specific isoform of KIF1A (NM_001244008.2, NP_001230937.1, isoform 1).

**Fig. 2:**
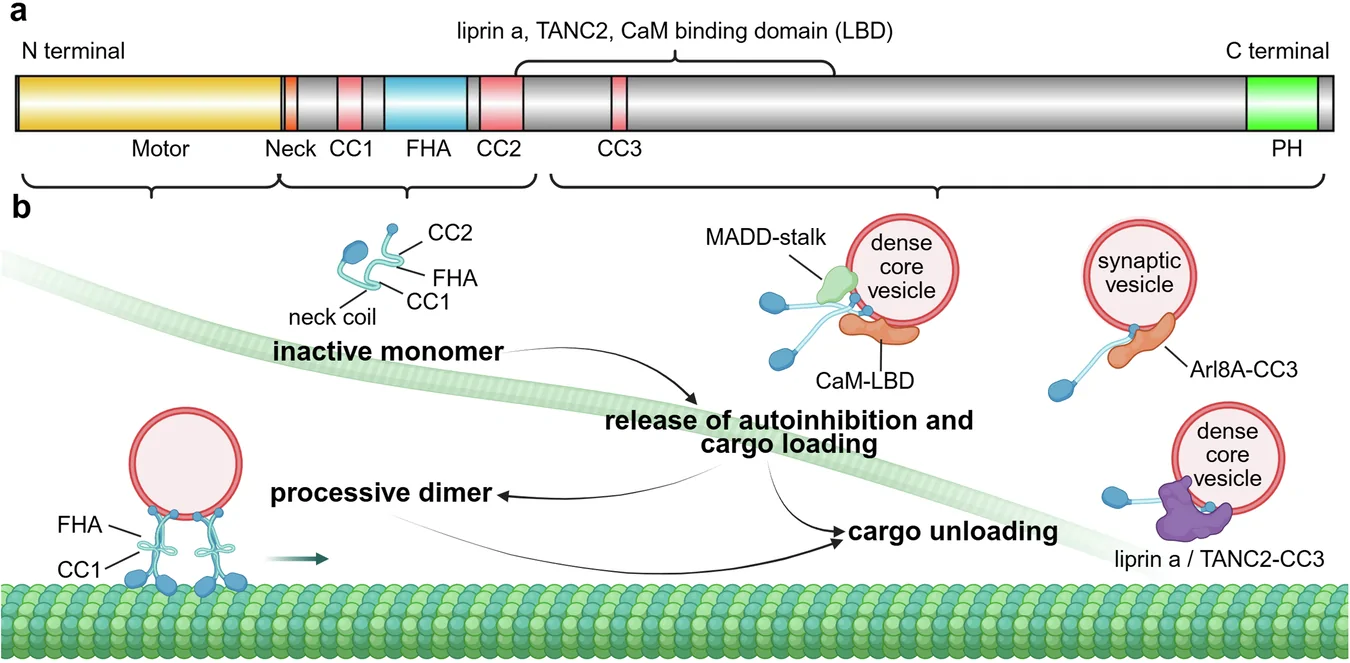
Characterised protein domains and functions in full-length KIF1A. **a** Schematic representation of the domain structure of full-length KIF1A with residue numbers based on the primary assembly isoform (NP_001230937.1, 1791 amino acids (aa)). Domains include MD motor domain (4-361 aa), NC neck coil (366-383 aa), CC1 coiled-coil 1 (438-471 aa), FHA forkhead associated domain (501-614 aa), CC2 coiled-coil 2 (631-690 aa), CC3 coiled-coil 3 (810-831 aa), PH pleckstrin homology domain (1673-1770 aa). According to Stucchi et al., residues 680-1113 form the binding region for liprin-alpha, TANC2 and calmodulin (CaM)-binding regions (LBD) [23]. The figure was generated using Illustrator of Biological Sequences (IBS) [64]. **b** Schematic of the KIF1A protein showing the transition from an inactive monomeric state to an active dimeric state upon binding with cargo, illustrating the process of autoinhibition release. The figure was not drawn to scale and was created in BioRender. Christodoulou (2025) https://BioRender.com/y61l493.

The function of KIF1A, like other kinesins in the Kinesin-3 family, is to transport cargoes over long distances and with high processivity towards the plus end of microtubules in an adenosine triphosphate (ATP)-dependent manner [10]. Structurally, the KIF1A protein functions in dimers, and one KIF1A comprises a head and neck region at the N-terminus and a tail domain at the C-terminus (Fig. 2a). The head domain is mostly responsible for the interaction with microtubules and ATP hydrolysis, affecting the affinity of binding, force generation and movement speed. The neck region, consisting of various coil-coiled (CC1, CC2, CC3) domains and a forkhead-associated (FHA) domain, interacts with itself and with other proteins to mediate protein activity and cargo recognition. Lastly, in the tail region, there is the pleckstrin homology (PH) domain, which is directly associated with cargo vesicles in neurons. Overall, the coordination of these structural domains enables efficient cargo transport along microtubules, with each domain playing a crucial role in regulating KIF1A’s motor function.

#### The head domain: navigating the microtubule

The head region of the KIF1A protein consists of a motor domain and a neck linker, which is essential for the protein’s function. This globular motor domain contains active sites for ATP hydrolysis and interactions with microtubules. The ATP hydrolysis centre, located at the N-terminus, is structurally organised with an alpha helix layer on the top and a beta-sheet layer beneath, supporting ATP catalysis. The microtubule-binding site lies at the C-terminus of the motor domain, consisting of additional alpha helices forming a supportive layer underneath [11]. Above the ATP hydrolyzation centre is loop L4 (P-loop), which creates a nucleotide-binding pocket for ATP binding and processing.

ATP hydrolysis triggers conformational changes in two key regions of the motor domain: switch I (helix a3, loop L9, B sheet B12) and switch II (loop L11, helix a4, loop L12, helix a5 and loop L13) [11]. The switch I region, located between P-loop and switch II, is responsible for catalysing the nucleophilic attack on the gamma-phosphate of ATP. Changes in the nucleotide state within the P-loop lead to conformational shifts in switch I, which in turn initiate rearrangements in salt bridges and subsequent conformational changes in the switch II region.

During microtubule binding, the positively charged lysine-rich loop L12 (K-loop) plays a crucial role by interacting with the negatively charged glutamate-rich C-terminal (E-hook) of tubulin. In a monomeric state, the weak electrostatic interaction serves as the key to facilitate ATP-independent biased diffusion [12]. In the highly processive dimeric state, conformational changes allow loop L12 to alternate binding with loop L11 in an ATP-dependent manner, supporting seamless movement along the microtubule [13].

The neck linker refers to the region between the motor domain and neck region, which is a structural element that docks onto the motor domain upon ATP binding, to enhance force generation and rapid movement along the microtubule [14]. In the ADP-bound state, it remains detached from the motor domain’s catalytic core.

#### The neck region: modulating KIF1A activity

The neck region begins with a short neck coil domain, which forms an alpha helix coil structure essential for KIF1A dimerisation [15]. The model proposed by Rashid et al. suggests that KIF1A is initially an inactive monomer (autoinhibited state) and becomes active through cargo-induced dimerisation. Upon cargo binding, intermolecular interactions assemble the neck coil, enabling dimerisation and activating KIF1A to move along microtubules in a hand-over-hand manner [16].

Following the neck coil, the KIF1A protein contains three short coiled-coil domains (CC1, CC2 and CC3), with an FHA domain positioned between CC1 and CC2. The CC1 region can fold back to form a helical bundle with the neck coil, preventing dimerisation and thus autoinhibiting KIF1A in its monomeric state [17].

#### The tail domain: facilitating cargo binding and delivery

KIF1A is responsible for delivering synaptic vesicle precursors and mature synaptic vesicles, lysosomes, DCVs and active zone precursor vesicles [1]. It transports essential proteins such as synaptophysin, synaptotagmin, Ras-related protein 3A (Rab3A), beta-secretase 1, Tropomyosin receptor kinase A (TrkA), low-density lipoprotein receptor and a-Amino-3-hydroxy-5-Methyl-4-isoxazolepropionic Acid Receptors (AMPAR) [18].

At the C-terminal end of the tail region lies the PH domain, which directly binds cargo vesicles through interactions with phosphatidylinositol-4,5-bisphosphate (PIP2) [19, 20].

The cargo-binding and transport capabilities of KIF1A can also be modulated by interactions between neck region domains and various adaptor proteins. For example, the FHA domain recognises phospho-threonine epitopes on other proteins, facilitating essential protein-protein interactions [21, 22].

The calcium-binding protein calmodulin also regulates cargo binding, increases calcium ion concentrations mediated via calmodulin, and enhances KIF1A binding to the transportation of DCVs [23].

### Variants in *KIF1A*

#### Variable clinical impact of *KIF1A* variants

Individuals with KAND display a wide range of clinical symptoms, with the same pathogenic variant in different individuals leading to significant variability in disease severity (Table 1).

**Table 1:** Summary of current reported *KIF1A* variants.

CDS	AAchange	Consequence	Inheritance	Zygosity	Recorded diagnosis	GOF or LOF	Domain
c.22 G > A	p.(Val8Met)	Missense variant	Familial	Heterozygous	HSP	GOF	MD
c.31 C > T	p.(Arg11Trp)	Missense variant	de novo	Heterozygous	Mild KAND, HSP		MD
c.32 G > A	p.(Arg11Gln)	Missense variant	de novo	Heterozygous	Mild KAND	LOF	MD
c.37 C > T	p.(Arg13Cys)	Missense variant	de novo	Heterozygous	Moderate KAND		MD
c.38 G > A	p.(Arg13His)	Missense variant	de novo	Heterozygous	Mild KAND, NESCAV		MD
c.52 C > T	p.(Arg18Trp)	Missense variant	de novo	Heterozygous	NR		MD
c.80 T > C	p.(Ile27Thr)	Missense variant	Familial	Heterozygous	HSP		MD
c.89 T > C	p.(Met30Thr)	Missense variant	de novo	Heterozygous	HSP		MD
c.110 T > C	p.(Ile37Thr)	Missense variant	de novo	Heterozygous	HSP		MD
c.160 T > G	p.(Tyr54Asp)	Missense variant	de novo	Heterozygous	Mild KAND		MD
c.167 A > G	p.(Tyr56Cys)	Missense variant	de novo	Heterozygous	HSP		MD
c.173 C > T	p.(Ser58Leu)	Missense variant	de novo	Heterozygous	Mild KAND, NESCAV	LOF	MD
c.206 C > T	p.(Ser69Leu)	Missense variant	Familial	Heterozygous	HSP		MD
c.218 T > G	p.(Val73Gly)	Missense variant	de novo	Heterozygous	HSP		MD
c.221 A > G	p.(Tyr74Cys)	Missense variant	de novo	Heterozygous	HSP		MD
c.232 G > A	p.(Gly78Ser)	Missense variant	de novo	Heterozygous	HSP		MD
c.254 C > A	p.(Ala85Asp)	Missense variant	de novo	Heterozygous	HSP		MD
c.265 T > G	p.(Tyr89Asp)	Missense variant	de novo	Heterozygous	Mild KAND	LOF	MD
c.274 T > C	p.(Cys92Arg)	Missense variant	de novo	Heterozygous	RTT	LOF	MD
c.275_276insAA	p.(Cys92*)	Frameshift variant	de novo	Heterozygous	RTT		MD
c.296 C > T	p.(Thr99Met)	Missense variant	de novo	Heterozygous	Severe KAND, PEHO, NESCAV	LOF	MD
c.304 G > A	p.(Gly102Ser)	Missense variant	de novo	Heterozygous	Moderate KAND, HSP	LOF	MD
c.305 G > A	p.(Gly102Asp)	Missense variant	de novo	Heterozygous	NESCAV	LOF	MD
c.308 A > C	p.(Lys103Thr)	Missense variant	de novo	Heterozygous	Moderate KAND		MD
c.308 A > G	p.(Lys103Arg)	Missense variant	de novo	Heterozygous	HSP		MD
c.317 C > A	p.(Thr106Asn)	Missense variant	de novo	Heterozygous	HSP		MD
c.326 G > T	p.(Gly109Val)	Missense variant	de novo	Heterozygous	NESCAV		MD
c.350 G > T	p.(Gly117Val)	Missense variant	de novo	Heterozygous	Mild KAND	LOF	MD
c.430 G > T	p.(Val144Phe)	Missense variant, splice region variant	de novo	Heterozygous	NESCAV		MD
c.442 G > A	p.(Glu148Lys)	Missense variant	de novo	Heterozygous	Severe KAND, HSP		MD
c.444 G > T	p.(Glu148Asp)	Missense variant	de novo	Heterozygous	NESCAV		MD
c.443 A > G	p.(Glu148Gly)	Missense variant	de novo	Heterozygous	NESCAV		MD
c.452 G > A	p.(Cys151Tyr)	Missense variant	de novo	Heterozygous	Mild KAND		MD
c.463 C > T	p.(Arg155Cys)	Missense variant	de novo	Heterozygous	Mild KAND		MD
c.467 A > T	p.(Asp156Val)	Missense variant	de novo	Heterozygous	Mild KAND		MD
c.470 T > A	p.(Leu157His)	Missense variant	de novo	Heterozygous	Severe KAND		MD
c.499 C > T	p.(Arg167Cys)	Missense variant	de novo	Heterozygous	HSP, NESCAV		MD
c.500 G > A	p.(Arg167His)	Missense variant	de novo	Heterozygous	HSP		MD
c.506 G > C	p.(Arg169Thr)	Missense variant	de novo	Heterozygous	NESCAV	LOF	MD
c.512 A > C	p.(His171Pro)	Missense variant	de novo	Heterozygous	Moderate KAND		MD
c.518 T > C	p.(LeuL173Pro)	Missense variant	de novo	Heterozygous	HSP		MD
c.532 G > A	p.(Val178Met)	Missense variant	de novo	Heterozygous	ALS		MD
c.536 A > G	p.(Glu179Gly)	Missense variant	de novo	Compound het	Severe KAND		MD
c.556 G > T	p.(Val186Phe)	Missense variant	de novo	Heterozygous	Severe KAND		MD
c.574 A > G	p.(Ile192Val)	Missense variant	de novo	Heterozygous	ALS		MD
c.595 G > A	p.(Gly199Arg)	Missense variant	de novo	Heterozygous	Mild KAND, HSP	LOF	MD
C.596 G > A	p.(Gly199Glu)	Missense variant	de novo	Heterozygous	Mild KAND		MD
c.604 G > C	p.(Ala202Pro)	Missense variant	de novo	Heterozygous	Mild KAND, NESCAV	LOF	MD
c.609 G > T	p.(Arg203Ser)	Missense variant, splice region variant	de novo	Heterozygous	Moderate KAND, NESCAV	LOF	MD
c.617 C > T	p.(Ala206Val)	Missense variant	de novo	Heterozygous	Mild KAND		MD
c.629 T > C	p.(Met210Thr)	Missense variant	Familial	Heterozygous	Mild KAND		MD
c.630 G > A	p.(Met210Ile)	Missense variant	de novo	Heterozygous	Mild KAND		MD
c.631 A > C	p.(Asn211His)	Missense variant	de novo	Heterozygous	Moderate KAND		MD
c.641 G > A	p.(Ser214Asn)	Missense variant	de novo	Heterozygous	Severe KAND		MD
c.643 A > C	p.(Ser215Arg)	Missense variant	de novo	Heterozygous	Severe KAND, NESCAV	LOF	MD
c.646 C > T	p.(Arg216Cys)	Missense variant	de novo	Heterozygous	Moderate KAND, NESCAV	LOF	MD
c.647 G > C	p.(Arg216Pro)	Missense variant	de novo	Heterozygous	NESCAV		MD
c.647 G > A	p.(Arg216His)	Missense variant	de novo	Heterozygous	Moderate KAND, HSP, NESCAV	LOF	MD
c.650 C > T	p.(Ser217Phe)	Missense variant	de novo	Heterozygous	NESCAV		MD
c.650 C > A	p.(Ser217Tyr)	Missense variant	de novo	Heterozygous	Moderate KAND	LOF	MD
c.664 A > C	p.(Asn222His)	Missense variant	de novo	Heterozygous	Autism		MD
c.685 C > T	p.(Arg229Cys)	Missense variant	de novo	Compound het	Moderate KAND		MD
c.715 G > A	p.(Glu239Lys)	Missense variant	de novo	Heterozygous	CMT		MD
c.726 C > G	p.(Ser242Arg)	Missense variant	Familial	Homozygous	HSP		MD
c.739 G > A	p.(Val247Met)	Missense variant	de novo	Heterozygous	Mild KAND		MD
c.743 A > G	p.(Asp248Gly)	Missense variant	de novo	Heterozygous	Mild KAND	LOF	MD
c.744 C > A	p.(Asp248Glu)	Missense variant	de novo	Heterozygous	RTT	LOF	MD
c.746 T > A	p.(Leu249Gln)	Missense variant	de novo	Heterozygous	NESCAV	LOF	MD
c.746 T > C	p.(Leu249Pro)	Missense variant	de novo	Heterozygous	Autism		MD
c.745 C > G	p.(Leu249Val)	Missense variant	de novo	Heterozygous	NESCAV		MD
c.751 G > A	p.(Gly251Arg)	Missense variant	de novo	Heterozygous	Severe KAND	LOF	MD
c.756 C > G	p.(Ser252Arg)	Missense variant	de novo	Heterozygous	HSP		MD
c.757 G > A	p.(Glu253Lys)	Missense variant	Both	Heterozygous	Severe KAND, NESCAV, PEHO	LOF	MD
c.760 C > T	p.(Arg254Trp)	Missense variant	de novo	Heterozygous	Severe KAND, NESCAV, HSP	LOF	MD
c.761 G > A	p.(Arg254Gln)	Missense variant	de novo	Heterozygous	Mild KAND, HSP, NESCAV	LOF	MD
c.761 G > C	p.(Arg254Pro)	Missense variant	de novo	Heterozygous	Mild KAND	LOF	MD
c.764 C > T*	p.(Ala255Val)	Missense variant	Familial	Homozygous	HSP	GOF	MD
c.764 C > A	p.(Ala255Asp)	Missense variant	Familial	Heterozygous	HSP		MD
c.773 C > T	p.(Thr258Met)	Missense variant	Both	Heterozygous	Mild KAND, HSP	LOF	MD
c.799 G > C	p.(Glu267Gln)	Missense variant, splice region variant	de novo	Heterozygous	Moderate KAND, NESCAV		MD
c.805 G > C	p.(Ala269Pro)	Missense variant	de novo	Heterozygous	HSP, NESCAV		MD
c.814 A > G	p.(Asn272Asp)	Missense variant	de novo	Heterozygous	NESCAV		MD
c.815 A > G	p.(Asn272Ser)	Missense variant	de novo	Heterozygous	Moderate KAND, HSP, NESCAV		MD
c.821 C > T	p.(Ser274Leu)	Missense variant	Both	Heterozygous	Severe KAND, HSP		MD
c.824 T > C	p.(Leu275Pro)	Missense variant	de novo	Heterozygous	Moderate KAND		MD
c.833 T > C	p.(Leu278Pro)	Missense variant	de novo	Heterozygous	Mild KAND, HSP	LOF	MD
c.835 G > C	p.(Gly279Arg)	Missense variant	de novo	Heterozygous	Mild KAND		MD
c.836 G > A	p.(Gly279Asp)	Missense variant	de novo	Heterozygous	Mild KAND		MD
c.839 A > G	p.(Lys280Arg)	Missense variant	de novo	Heterozygous	Moderate KAND		MD
c.914 C > T	p.(Pro305Leu)	Missense variant	de novo	Heterozygous	Severe KAND, HSP, RTT	LOF	MD
c.917 A > G	p.(Tyr306Cys)	Missense variant	de novo	Heterozygous	Severe KAND		MD
c.919 C > G	p.(Arg307Gly)	Missense variant	de novo	Heterozygous	Moderate KAND, NESCAV		MD
c.920 G > A	p.(Arg307Gln)	Missense variant	de novo	Heterozygous	Severe KAND, HSP, NESCAV	LOF	MD
c.920 G > C	p.(Arg307Pro)	Missense variant	de novo	Heterozygous	Moderate KAND	LOF	MD
c.939 G > T	p.(Trp313Cys)	Missense variant	Familial	Heterozygous	HSP		MD
c.941 T > C	p.(Leu314Pro)	Missense variant	de novo	Heterozygous	Mild KAND		MD
c.946 C > T	p.(Arg316Trp)	Missense variant	Both	Heterozygous	Severe KAND, HSP, NESCAV	LOF	MD
c.947 G > A	p.(Arg316Gln)	Missense variant	de novo	Heterozygous	Mild KAND		MD
c.962 G > A	p.(Gly321Asp)	Missense variant	Familial	Heterozygous	HSP		MD
c.967 T > C	p.(Ser323Pro)	Missense variant	de novo	Heterozygous	Mild KAND		MD
NR	p.(Thr341Pro)	Missense variant	de novo	Heterozygous	HSP		MD
c.1031 C > T	p.(Thr344Met)	Missense variant	de novo	Heterozygous	Moderate KAND	LOF	MD
c.1048 C > G	p.(Arg350Gly)	Missense variant	de novo	Homozygous	HSP	GOF	MD
c.1048 C > T	p.(Arg350Trp)	Missense variant	de novo	Heterozygous	HSP		MD
c.1108 C > T	p.(Arg370Cys)	Missense variant	de novo	Heterozygous	ALS		NC
c.1139 G > C	p.(Arg380Pro)	Missense variant	Familial	Heterozygous	HSP		NC
c.1190 C > A	p.(Ala397Asp)	Missense variant	Familial	Heterozygous	NESCAV	GOF	-
c.1214dup	p.(Asn405Lysfs*40)	Frameshift variant	de novo	Compound het	HSP		-
c.1217 C > A	p.(Ala406Asp)	Missense variant	de novo	Heterozygous	Moderate KAND		-
c.1271 C > T	p.(Pro424Leu)	Missense variant	de novo	Heterozygous	ALS		-
c.1406 C > G	p.(Ala469Gly)	Missense variant	de novo	Heterozygous	HSP		CC1
c.1451 C > A	p.(Ala484Asp)	Missense variant	de novo	Heterozygous	Moderate KAND		-
c.1463 A > T	p.(Asp488Val)	Missense variant	de novo	Compound het	Severe KAND		-
c.1856C > G	p.(Ala619Gly)	Missense variant	de novo	Heterozygous	ALS		-
c.1894C>T	p.(Gln632*)	Stop gained	de novo	Heterozygous	HSP		CC2
c.2131 C > T	p.(Arg711Trp)	Missense variant	de novo	Heterozygous	ALS		LBD
c.2423 A > G	p.(Tyr808Cys)	Missense variant	de novo	Heterozygous	ALS	GOF	LBD
c.2554 C > T	p.(Arg842Cys)	Missense variant	de novo	Heterozygous	HSP		LBD
c.2658del	p.(Ser887Profs*64)	Missense variant	Familial	Homozygous	HSAN II		LBD
c.2751_2753delGGA	p.(Glu917del)	Frameshift variant	Familial	Homozygous	Mild KAND		LBD
c.2753_2754insGGA	p.(Asp918delinsGluAsp)	Frameshift variant	Familial	Heterozygous	ALS		LBD
c.2840delT	p.(Leu947Argfs*4)	Frameshift variant	de novo	Homozygous	HSAN II		LBD
c.2880 C > A	p.(Asn960Lys)	Missense variant	de novo	Heterozygous	HSP		LBD
c.2909 G > A	p.(Arg970His)	Missense variant	de novo	Compound het	HSP		LBD
c.2935 G > A	p.(Glu979Lys)	Missense variant	de novo	Heterozygous	ALS		LBD
c.3247 G > A	p.(Ala1083Thr)	Missense variant	Familial	Heterozygous	ALS		LBD
c.3380 T > C	p.(Ile1127Thr)	Missense variant	Familial	Heterozygous	HSP		-
c.3533 T > C	p.(Phe1178Ser)	Missense variant	de novo	Heterozygous	ALS		-
c.3601 C > T	p.(Arg1201Cys)	Missense variant	de novo	Heterozygous	ALS	GOF	-
c.3617 G > A	p.(Arg1206Gln)	Missense variant	de novo	Heterozygous	ALS		-
c.3680 C > T	p.(Pro1227Leu)	Missense variant	de novo	Heterozygous	ALS		-
c.3719dupA	p.(Asn1240Lysfs*2)	Frameshift variant	de novo	Homozygous	HSP		-
c.3763 G > A	p.(Val1255Met)	Missense variant	de novo	Heterozygous	ALS		-
c.3871 C > T	p.(Arg1291Cys)	Missense variant	Familial	Compound het	HSAN II		-
c.3887 G > A	p.(Arg1296His)	Missense variant	Familial	Heterozygous	HSP		-
c.3898 G > A	p.(Val1300Met)	Missense variant	Familial	Compound het	HSAN II		-
c.4096_4103dup	p.(Asp1369fs)	Frameshift variant	de novo	Heterozygous	HSP		-
c.4278 C > G	p.(Tyr1426*)	Frameshift variant	de novo	Heterozygous	HSP		-
c.4370 G > A	p.(Arg1457Gln)	Missense variant	de novo	Heterozygous	ALS	Conflicting	-
c.4594delC	p.(Pro1532Argfs*67)	Frameshift variant	de novo	Heterozygous	HSP		-
c.4682 C > T	p.(Thr1561Met)	Missense variant	de novo	Heterozygous	ALS		-
c.4771 C > T	p.(Arg1591Trp)	Missense variant	de novo	Compound het	Moderate KAND		-
c.4778 C > T	p.(Pro1593Leu)	Missense variant	de novo	Heterozygous	ALS		-
c.4927 G > A	p.(Asp1643Asn)	Missense variant	de novo	Heterozygous	ALS		-
c.4955 G > A	p.(Arg1652Gln)	Missense variant	de novo	Heterozygous	ALS		-
c.5046 C > A	p.(Tyr1682fs)	Frameshift variant	de novo	Heterozygous	HSP		PH
c.5063 C > T	p.(Pro1688Leu)	Missense variant	de novo	Heterozygous	ALS	Conflicting	PH
c.5131 G > A	p.(Asp1711Asn)	Missense variant	de novo	Heterozygous	ALS		PH
c.5150 G > T	p.(Arg1717Leu)	Missense variant	de novo	Heterozygous	ALS		PH
c.5231 C > T	p.(Ala1744Val)	Missense variant	de novo	Heterozygous	ALS	GOF	PH
c.5271dupC	p.(Ser1758Glnfs*7)	Frameshift variant	de novo	Compound het	HSAN II		PH

In addition, the severity of symptoms in KAND has been found to correlate with the locations of the variants within the *KIF1A* gene. The cohort study by Boyle et al. of KAND individuals revealed that most variants were clustered in the N-terminal region, with 111 out of 114 individuals (97.4%) carrying variants in the highly conserved motor domain, crucial for microtubule binding and ATP-dependent movement of KIF1A [24]. In this study, individuals with variants, particularly affecting ATP hydrolysis or microtubule binding (such as the P-loop, switch I, and switch II), exhibited more severe clinical symptoms. Furthermore, functional analyses have also supported this correlation. For instance, the p.(Thr99Met) and p.(Glu253Lys) variants impair ATP hydrolyzation, and the p.(Pro305Leu) variant, located in loop L12 responsible for direct microtubule binding, were among the variants leading to the most severe disease phenotypes in KAND [3, 25, 26]. In contrast, variants p.(Arg167Cys) or p.(Arg167His) located in loop L8, outside of the ATP or microtubule binding motifs, resulted in milder symptoms such as lower limb spasticity only [26–28]. In addition, the mean age of onset reported in Boyle et al. was 11 months, with a range spanning from birth to 9 years [24]. In contrast, among patients diagnosed with ALS carrying *KIF1A* variants, 36 out of 41 individuals were heterozygous for variants in the C-terminal regions, specifically downstream of the CC2 domain [29]. These individuals exhibited a mean disease onset of 56.7 years, ranging from 40 to 67 years, and exhibited marked disease severity. Given that the C-terminal domains are primarily responsible for cargo binding and transport, it is hypothesised that the missense variants in these regions may selectively disrupt specific types of cargo transport. Consequently, this selective impairment might progressively hinder cellular function over time, contributing to the later onset of disease phenotypes as the cumulative impact of cargo delivery becomes more pronounced.

In addition, homozygous or biallelic variants in C-terminal regions, such as p.(Leu947Argfs*4) and p.(Ser1758Glnfs*7), were found to be associated with HSAN IIC [30]. Patients with these variants exhibited relatively early disease onset, with a mean age of 8.5 years (ranging from birth to 15 years), compared to ALS patients with heterozygous missense C-terminal variants. It is likely that the biallelic or compound heterozygous nature of these variants results in the near complete absence of functional KIF1A proteins, likely contributing to severe neurological outcomes at an early age.

Heterozygous variants identified in both KAND and ALS are likely to exert a dominant negative effect, impacting coordinated cargo delivery. This suggests that a heterodimer formed by a functional and a variant KIF1A is likely to malfunction either in microtubule binding, ATP hydrolysis or cargo association, depending on the specific location of the variant within the protein.

On the other hand, heterozygous variants p.(Gln632*) and p.(Tyr1426*) found in individuals with pure SPG30 led to relatively earlier disease onset in patients (at 30 years and <10 years respectively) but with milder symptoms and slower disease progression compared to homozygous or compound heterozygous variants found in HSAN IIC [28, 31]. This leads to the hypothesis that at least some *KIF1A* variants could also lead to a disease phenotype through a haploinsufficiency mechanism. However, it is of note that currently, no studies investigating RNA and protein expression levels of wild type and variant KIF1As have been conducted, so it remains unclear how the nonsense variants contribute to disease.

#### Variant impact on protein functionality

Due to the complex inheritance patterns and diverse variant locations across different protein domains, the impact of a *KIF1A* variant should be assessed on an individual basis.

### Variants in the motor domain

The KIF1A protein functions as an active dimer, where missense variants in the motor domain have been demonstrated through functional analyses to contribute to disease phenotypes via a dominant-negative mechanism [32–34]. The movement of KIF1A along the microtubule network occurs in a ‘hand-over-hand’ pattern, necessitating the full functionality of both KIF1A monomeric motors. Consequently, a single variant allele in heterozygous individuals could theoretically compromise up to 75% of the KIF1A dimeric motors produced, rendering them dysfunctional. Moreover, variants in the motor domain are associated with delays in all forms of cargo transport, as they disrupt the foundational mechanisms required for effective transport.

**Fig. 3:**
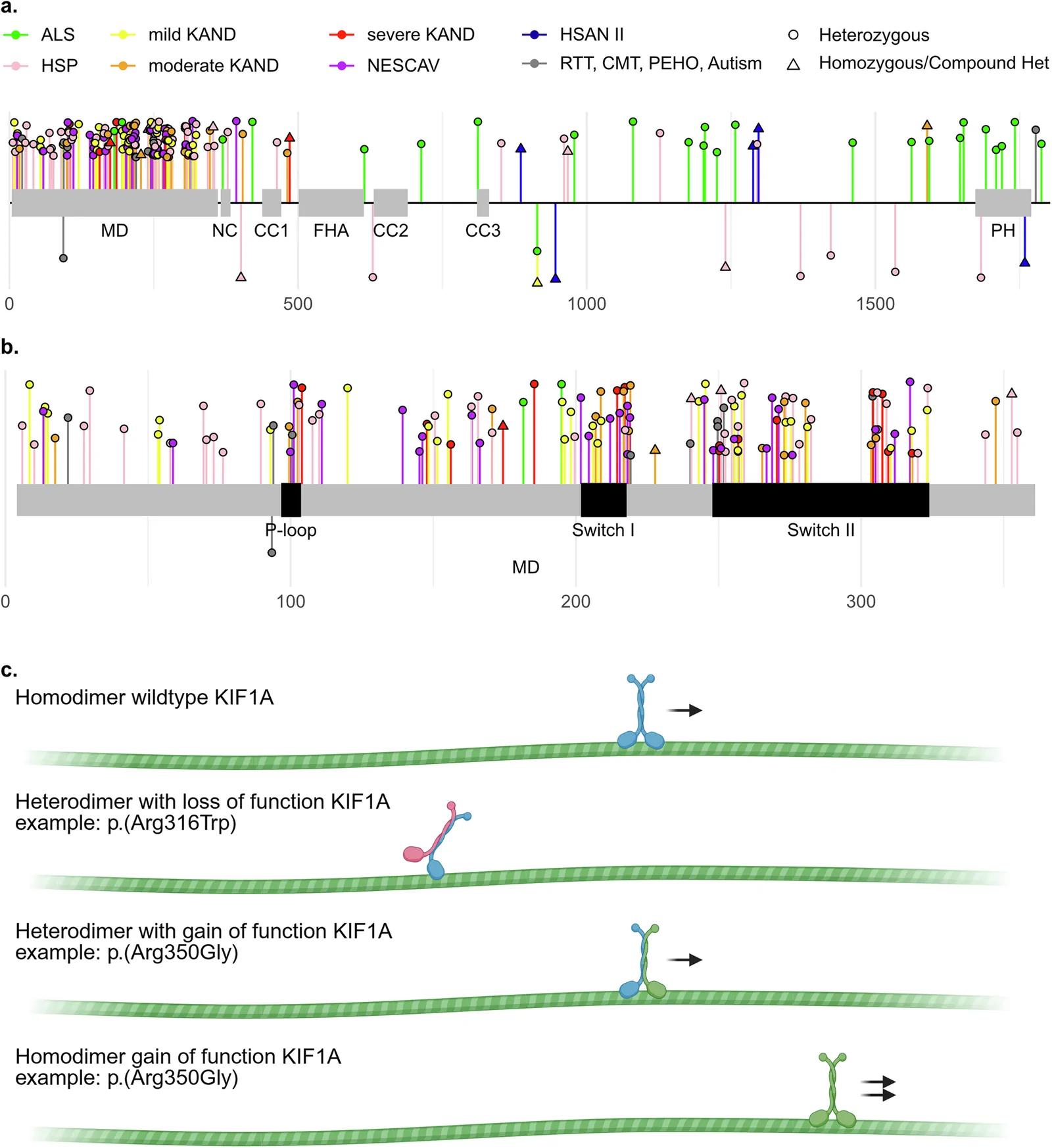
*KIF1A* variants in relation to the protein structure. **a** Lollipop chart of literature-reported *KIF1A* variants. For each variant, round lollipop heads represent heterozygous variants, triangle heads represent homozygous and compound heterozygous variants. Variants above the protein schematic are missense variants, variants below the schematic are frameshift and nonsense variants. Green: Amyotrophic Lateral Sclerosis (ALS), pink: hereditary spastic paraplegia (HSP), yellow: mild KAND, orange: moderate KAND, red: severe KAND, purple: NESCAV syndrome, blue: HSAN type II, grey: other neurodevelopmental disorders (Rett, Charcot-Marie Tooth, PEHO Syndrome, Autism spectrum). Figure created by R Studio. **b** Lollipop chart of KIF1A variants in the motor domain. **c** The function of the KIF1A motor is determined by the monomer with less mobility. Schematic representation of the hypothesised speed of different KIF1A dimers (two wild type, wild type + loss of function, wild type + gain of function, gain of function + gain of function). The less processive KIF1A monomer will be the rate limiter for any dimer in which it participates. The figure was not drawn to scale and was created in BioRender. Christodoulou (2025) https://BioRender.com/dhr5qbeusing.

On the other hand, for gain-of-function missense variants in the motor domain, such as p.(Ala255Val) and p.(Arg350Gly), the inheritance of two alleles (in homozygous individuals) is required to result in a disease phenotype [35, 36]. This suggests that the function of a KIF1A dimer is determined by the monomer with the lower capacity for microtubule movement (Fig. 3b). In other words, the less processive KIF1A monomer will be the rate limiter for any dimer in which it participates. Heterodimers consisting of a loss-of-function monomer dimerised with a wild-type monomer will be dysfunctional, while a heterodimer of a gain-of-function motor dimerised with a wild-type motor will perform normal functions instead of resulting in a ‘hyperactive’ or ‘superprocessive’ motor.

Shewale et al. grouped the variants in the motor domain into different categories based on protein performance in functional studies [37]. For instance, refer to those variants that do not impede the KIF1A protein from reaching the peripheral region of cells. **Microtubule-binding variants** (also referred to as ‘rigour’ variants) display line patterns around the nucleus, indicating that these variant KIF1A proteins can associate with microtubules but have lost the ability to hydrolyse ATP molecules and traverse the microtubule. In silico protein modelling results also agreed with the experimental findings, as these variants were all located in motifs responsible for ATP binding, hydrolysation and release. Clinically, most variants in this category lead to a severe KAND phenotype [24]. **Cytoplasmic variant** proteins cannot bind to microtubules, leading to a diffused pattern in the cell body rather than a line pattern around the nucleus. Most variants in this category were associated with KAND of mild to moderate severity or NESCAV phenotype. Interestingly, the other gain-of-function variant, p.(Arg350Gly), also belonged to this group, suggesting that the resulting protein was unable to travel to the peripheral region when introduced to a constitutively active KIF1A (1-393) gene construct and transfected in COS-7 cells. This result was in contrast to a single molecule assay using full-length human KIF1A, which found that the landing rate and velocity of p.(Arg350Gly) KIF1A motors were significantly increased compared to wild type [38]. Given the proximity of variants p.(Arg350Gly) to the neck region responsible for protein activity, a truncated constitutively active motor might not be suitable to study the gain-of-function variants in the motor domain, as the wild-type truncated motor has already been modified to be super-processive. Thus, various experimental methods may need to be explored to study the variants and gain a more complete insight into the impact of variants on protein function.

Previously, most functional assays undertaken to analyse the effect of *KIF1A* variants were conducted by transfecting a truncated *KIF1A* plasmid with just the motor domain and neck linker into stable cell lines and analysing protein motility. However, this assay does not provide a complete picture of the behaviour of variant KIF1A in true neuronal environments. Additionally, pathogenic *KIF1A* variants are typically heterozygous, and a recent study by Rao et al. [39] found that heterodimers of wild-type and KAND-specific variant KIF1A targeting the motor domain at amino acid position Arg216 and Arg307 showed reduced motility compared to wild-type homodimers, but not as severe as variant homodimers. In addition, findings from direct protein functional analysis of variant homodimers correlate strongly with clinical severity in KAND patients, thus placing homodimers as the key driver of disease progression, while heterodimers serve to preserve the baseline function in presynaptic transport [39]. Future research should also investigate the true ratio of wild-type homodimer, heterodimer and variant homodimer in patient-specific neurons to examine how differently configured dimers might contribute to disease mechanisms and clinical phenotypes in KAND.

### Variants towards the stalk and tail regions

Between the N-terminal motor and C-terminal cargo-binding domains, KIF1A neck and stalk domains play a role in regulating autoinhibition and dimerisation. Classifying pathogenicity of these variants in these regions can be more challenging, in part because structural consequences are more difficult to assess in these comparably unstructured domains.

Not only do motor domain missense variants lead to spastic paraplegia. Frameshift and nonsense variants in the C-terminal domain have also been reported in HSP patient cohorts, suggesting that haploinsufficiency could also be a pathogenic mechanism [28]. When compared with other variant types, these variants are associated with earlier disease onset (all <10 years) compared to heterozygous missense variants in the same region (ALS, age of onset >23, mostly >50 years) but lead to milder clinical manifestations (only lower limb spasticity and weakness, normal cognition) compared to missense dominant-negative motor domain variants. However, no functional analyses have been performed to elucidate the impact of these haploinsufficient variants on the structure and function of KIF1A, which may either lead to nonsense-mediated decay or the production of dysfunctional proteins. In addition, heterozygous *Kif1a* knockout mice were found to develop neuropathy at later stages (after 5–6 months), suggesting that haploinsufficiency also contributes to the KAND disease mechanism [40]. Functional assays exploring the endogenous level of wild-type KIF1A monomers versus variant KIF1A monomers could be conducted to confirm whether variants also lead to haploinsufficiency.

Surprisingly, missense variants in the C-terminal regions found in ALS patient cohorts led to a gain-of-function instead of hindering cargo binding. While the gain-of-function variants in the motor domain seemed to be well tolerated in heterozygous individuals, the heterozygous C-terminal variants were shown to enhance cargo transport significantly. Functional analysis has revealed that the p.(Tyr808Cys) variant increased binding affinity to synaptophysin, while p.(Arg1201Cys), p.(Arg1457Gln), p.(Pro1688Leu), and p.(Ala1744Val) variants showed increased binding to Rab3A and vesicle-associated membrane protein 2 (VAMP2) — key markers of synaptic vesicle precursors—when co-transfected into HEK293 cells and cultured cortical neurons [29]. Another hypothesis would be that increased binding affinity disrupts the ability for KIF1A to release cargo in a coordinated response to signals at the axon terminals, such that cargo delivery itself is still compromised. These findings underscore the critical need to maintain a balance in axonal transportation, as either delays in or enhanced cargo delivery can lead to clinical manifestations detrimental to neuronal development and survival.

However, contradictory results were subsequently observed by the same group, in patient-derived iPSCs, as fluorescence microscopy showed that both p.(Arg1457Gln) and p.(Pro1688Leu) variant KIF1A proteins and synaptophysin accumulated significantly in the proximal regions of differentiated motor neurons, suggesting the variants in the C-terminal region might impact protein motility and subsequent cargo delivery in iPSCs [41]. Thus, it remains crucial to assess the impact of the variant on protein function in different model systems and to continue exploring more convenient assays to functionally analyse the variants.

### Current treatments for KAND

#### Managing symptoms

At present, there are limited therapeutic options and no cure for individuals with KAND, and current treatment strategies remain limited. The management primarily focuses on alleviating symptom through supporting interventions such as physiotherapy and occupational therapy, and in some cases, pharmacological and surgical approaches, including baclofen, Botox injections and selective dorsal rhizotomy [6, 42]. The early interventions, including speech therapy and behaviour therapy, also assist capacity building and improve the lives of KAND individuals.

### Spasticity

KAND patients utilise a wide variety of physiotherapy interventions to manage spasms, motor skills, and muscle tone dysregulation [43]. Because KAND is neurodegenerative, these interventions are leveraged as maintenance therapies that develop skills but also counteract progressive loss of function.

Patients experiencing spasticity rely heavily on baclofen, a muscle relaxant inhibiting neuronal activity in the spinal cord to prevent muscle contractures, and injections of Botox, which reduces peripheral responses in targeted muscle groups. These therapies partially address spasticity, but KAND is a progressive disorder in which neurodegeneration leads to muscle weakness that limits mobility. Ultimately, many patients rely on assistive mobility devices, including crutches, walkers, and wheelchairs.

### Epilepsy

Epilepsy is variable in KAND, with absence, tonic-clonic, infantile spasms, and atonic seizures being the most commonly reported. KAND patients often experience epileptiform activity in the form of spikes during slow-wave sleep. Levetiracetam, clobazam and lamotrigine are commonly used antiseizure medications, and 43% of patients need multiple antiseizure medications [8, 44].

### Peripheral neuropathy

Pain is a complicated feature of KAND, experienced by 32% of KAND patients, including reduced pain sensitivity, putting them at risk of aggravated injuries [45]. In addition, families with missense, motor domain variants also report neuropathic pain in extremities, which are sometimes treated with gabapentin. Peripheral neuropathy may also impact proprioception, temperature regulation, bladder control, and symptoms with no KAND-informed treatments.

### Emerging treatments for KAND

#### RNA modification: antisense oligonucleotide (ASO) therapy

Given the progressive nature of KAND and its variable onset observed among affected individuals, developing a strategy to slow disease progression or delay disease onset remains crucial and could serve as a bridging approach before the discovery of a cure for KAND. Due to the early onset and severity of dominant-negative variants, the use of an ASO to knock down variant *KIF1A* expression could have an ameliorating effect on the phenotype, mitigating the disease’s impact and providing a window of time to develop more definitive treatments.

A recent study by Ziegler et al. [46] reported a 9-year-old female with KAND who carried a heterozygous motor domain variant, p.(Pro305Leu). This KAND individual was treated with an individualised ASO (nL-KIF1-001), developed in collaboration with the n-Lorem Foundation (https://www.nlorem.org/), which selectively down-regulated the expression of the variant *KIF1A* allele, reducing the levels of variant KIF1A homodimer and heterodimers [46]. A decrease in paroxysmal episodes was reported by the parents, which was associated with improvements in the EEG and a reduced frequency of epileptiform spikes. The frequency of falls experienced by the child improved, accompanied by an increase in motor skills, physical activities and ambulation. Similar improvements were also observed in speech and attention.

Despite these promising results with the personalised KIF1A ASO therapy, there are still unmet needs. Clinical assessments of the ASO-treated KAND individual showed minimal differences in the distance walked and cognitive performance, and only mild improvements in fine and gross motor function. However, while the proband’s overall cognition remained stable during the initial treatment period, whether it will have a long-term positive impact on the degenerative course of the disease remains to be seen. In addition, other multi-system manifestations, including peripheral neuropathy, gastroesophageal reflux, optic nerve atrophy and behavioural outbursts, were not influenced by intrathecal injections of the ASO. Multiple factors may contribute to this, including lack of access to optic and peripheral systems by intrathecal administration, but also pre-existing unsalvageable degeneration in affected circuits. Lastly, based on our understanding of the pathophysiology of KAND, knocking down half of *KIF1A* expression could lead to disease features through a haploinsufficiency mechanism, with a likely later onset but probably milder symptoms. As such, ASO treatment primarily serves as a bridging approach, mitigating the pathological effects of the variant and slowing disease progression rather than providing a definitive cure.

#### Neurotransmitter replacement therapy

Biogenic amines are classical neurotransmitters that are found deficient in many neurological disorders. In a clinical trial conducted by Van Karnebeek et al., a patient carrying the *KIF1A* variant p.(Ala202Pro) was treated with levodopa/carbidopa and 5-hydroxytryptophan (5HTP) and showed improvements in tremor, myoclonus, mood, sleep and behaviours [47]. Although the exact pathophysiology behind KAND and secondary biogenic amine deficiencies remain unclear, it was well known that the KIF1A protein is responsible for the delivery of synaptic vesicles along with their precursors and DCVs, which are filled with neurotransmitters to be released at the synapse. Thus, neurotransmitter replacement therapy may serve as a potential treatment for KAND patients with loss-of-function variants, targeting the downstream effects of KIF1A deficits.

### Potential therapies for KAND

**Fig. 4:**
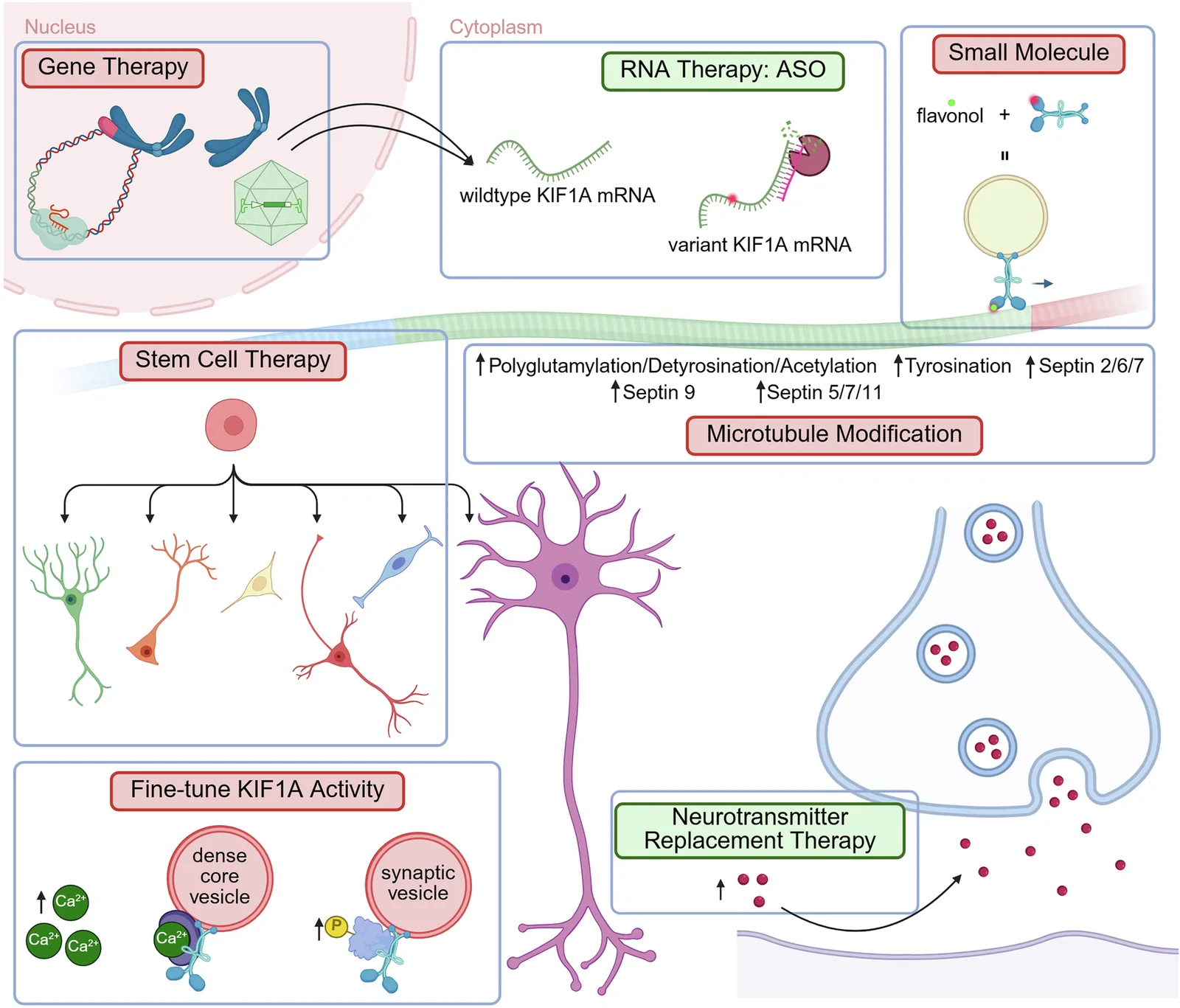
Available and prospective treatment strategies for KAND. This figure illustrates the multiple approaches for the development of treatment strategies for *KIF1A*-Associated Neurological Disorder (KAND). Emerging treatment strategies are shown in green title boxes. Potential treatment strategies are shown in red title boxes. The figure was not drawn to scale and was created in BioRender. Christodoulou (2025) https://BioRender.com/qnl458a.

While therapies targeting the underlying genetic causes of KAND are progressing, current treatment approaches primarily focus on managing symptoms and slowing disease progression, with ASOs offering the most promising, albeit partial, benefits. Ongoing research and more extensive clinical trials will be needed to explore further therapeutic avenues to provide more effective treatments for KAND patients (Fig. 4).

#### Fine-tuning KIF1A activity

As indicated above, both reduced and enhanced functions of KIF1A will result in disease phenotypes. Thus, the protein activity of KIF1A could be fine-tuned to a healthy level as a potential treatment for KAND.

Heterozygous KAND individuals have some level of wild-type KIF1A neuronal protein expression. For those with variants that cannot form functional dimers, the disease phenotype is due to insufficient production of and subsequent cargo transport by the wild-type functional KIF1A proteins. As heterodimers of wild type and variant KIF1A show some level of protein functionality, whereas homodimers of variant KIF1A loses function completely, overexpression of wild-type KIF1A to outnumber variant KIF1A, leading to the production of more wild-type homodimers or heterodimers, may boost cargo transport in patients with missense dominant-negative motor domain variants. One way to treat this mechanism would be to upregulate the wild-type KIF1A activity, either by increasing the expression of wild-type KIF1A or modulating the wild-type KIF1A to transport more cargoes faster. For instance, the regulator of KIF1A, calmodulin, was found to increase the loading of DCVs onto KIF1A and boost subsequent transport motility [23]. In calcium-supplemented conditions, a significant clustering of KIF1A at the synapses and increased motility of KIF1A-neuropeptide Y complexes, indicative of DCV transport, were observed minutes after bicuculline treatment, a molecule that enhances neuronal activity by inhibition at the γ-aminobutyric acid receptor. This suggests that targeting calcium levels in the neurons that interact with calmodulin, thereby increasing DCV transport by KIF1A, could help to boost the activity of wild-type KIF1A in heterozygous patients, potentially reducing the neurological consequences of delay in cargo transport, which in turn could potentially slow disease progression.

Another major cargo for KIF1A is the synaptic vesicle precursor, whose transport was found to be regulated by Huntingtin [48]. The phosphorylation of Huntingtin was found to increase the accumulation of synaptic vesicles at synapses by recruiting KIF1A to VAMP2, a major protein found on synaptic vesicles. In addition, homozygous constitutively phosphorylated Huntingtin mice (*Htt*^*S421D/S421D*^) showed impaired motor learning skills, similar to the clinical symptoms encountered by KAND patients carrying gain-of-function variants. Transiently reducing *KIF1A* expression in these mice improved their motor learning within a few days. Thus, it could be possible to fine-tune cargo transport in neurons and maintain synapse homeostasis, to potentially reverse the impact on motor skills in KAND patients. Similar approaches have been tested in Rett syndrome, as promoting phosphorylation of Huntingtin by a calcineurin inhibitor FK506 restores axonal transport of BDNF-carrying signalling endosomes, and improved behavioural phenotypes and survival in a Rett syndrome mouse model (*Mecp2*^*−/y*^ male hemizygous mouse) [49]. Since it is predicted that N-terminal *KIF1A* variants might disrupt the fundamental basis of cargo transport, the ability for KIF1A to move along microtubules, whereas C-terminal variants might only affect one type of cargo transport, a single target could be selected for C-terminal variant proteins specifically, depending on which type of cargo transport the variant disrupts, or a combination of the above strategies could be employed to specifically enhance all types of cargo transport for N-terminal variants.

For gain-of-function variants that give rise to hyperactive KIF1A motors, possible inhibitory processes that could decrease KIF1A activity could be targeted. For instance, a common anaesthetic drug, propofol, was found to decrease kinesin-mediated cargo transport by 25–60% in hippocampal neurons, specifically affecting the velocity, run length, and the association of KIF1A with synaptic vesicle protein VAMP2 [50].

Another potential mechanism to regulate KIF1A activity is by modulating the microtubular ‘highway’ for cargo transport. These strategies could impact on the interaction between the motor domain of KIF1A and the microtubule, benefiting the transport efficiency of N-terminal variants. Polyglutamylation, a form of tubulin C-terminal posttranslational modification, which interacts with the K-loop in KIF1A, was found to significantly increase the run length, landing rate and pause frequency, and duration associated with the super-processive motor [51]. It was previously shown that polyglutamylation of microtubules is also associated with further detyrosination [52], thus, mechanisms that decrease glutamylation and detyrosination of microtubules could be employed to halt the KIF1A super-processive transport, caused by gain-of-function variants. Conversely, pathways that increase the levels of these microtubular posttranslational modifications could be targeted for loss-of-function variants. Interestingly, contradictory results were observed by Tas et al., reporting that kinesin-1 proteins favour detyrosinated and acetylated microtubules in their cargo transport, whereas kinesin-3 motors (including KIF1A) favour microtubule modifications opposite to kinesin-1 proteins, i.e., tyrosinated and deacetylated microtubules [52].

Microtubule-associated septin complexes can modulate protein trafficking by interacting with different motor proteins, including KIF1A [53]. Specifically, the septin 2/6/7 complex can inhibit the KIF1A motor by hindering the ‘stepping’ motion along microtubules. However, the addition of septin 9 to the septin 2/6/7 complex or septin 9 alone can dampen this inhibitory effect. Similarly, the septin 5/7/11 complex also enhances the motility of the KIF1A protein, mimicking the function of septin 9. These findings suggest that modulating microtubule decorations with specific septin complexes could be a promising therapeutic strategy for KAND patients, depending on whether the goal is to enhance or suppress KIF1A activity.

#### Drug screening

Different model systems and organisms developed in the KIF1A research field have provided valuable insights into the pathophysiology of KAND and can serve as great tools for drug screening and testing.

One such model, *C. elegans*, is particularly well-suited for developmental biology studies. The development of *C. elegans* models allowed researchers to explore intragenic and extragenic suppressors that could potentially restore function to patient-specific *KIF1A* variants. A notable suppressor screen approach in this context involves creating double-mutant worms by introducing new random mutations and screening for those that rescue the initial patient variant. While this method does not directly translate to treatment for patients, it provides valuable insights for developing drug candidates. For instance, introducing the p.(Asp177Asn) variant into the *unc-104* sequence restored function to the motor domain variant p.(Arg251Gln) [34]. The double-mutant worms displayed better motility, had increased synaptic vesicle delivery to the dorsal synaptic region, while double-mutant proteins displayed improved velocity, landing rate and run length comparable to wild type. Another screen on the *C. elegans* carrying the loss-of-function variant p.(Asp1497Asn), located in the PH domain and which causes decreased binding of unc-104 to PIP2, identified two intragenic suppressors, variants p.(Asp948Asn) and p.(Ala950Val), both located in the stalk domain [54]. This evidence points to a possible self-interacting mechanism of the unc-104 protein between the stalk region and the PH domain, offering new insights into its homologue KIF1A′s protein structure and how modifying one domain can potentially correct defects in another domain within KIF1A.

Furthermore, Chai et al. observed that the p.(Arg11Gln) mutant in *C. elegans* spontaneously acquired nutrients during starvation, leading to significant improvements in abnormalities of their body morphology [55]. Subsequent experiments showed that it was the plant flavonol, fisetin, present in *C. elegans* feeding nutrients, which can associate with the groove near the ATP binding pocket affected by p.(Arg11Gln) variant and induced a structural change that reversed the impact of the variant residue. The use of this small molecule that directly targets the variant functional motif appeared to specifically restore the function of heterodimers and variant KIF1A homodimers instead of eliminating them and triggering a haploinsufficiency mechanism. Although this finding is specific to the p.(Arg11Gln) variant, given the high conservation between the *unc-104* gene and *KIF1A*, it strongly supports the use of *C. elegans* as a robust and efficient model organism for future suppressor and drug screening studies in KAND, potentially benefiting a broader population affected with a diverse range of variants in KAND.

#### Stem cell-based therapy

It is notable that KIF1A deficiency leads to marked neuronal degeneration and death in both mouse models and cultured neurons [10]. Thus, the use of cell replacement therapy, which directly replenishes the lost neurons, could serve as a potential treatment, thereby relieving some neurological defects in affected individuals. Currently, the only stem cell-based therapy that has been routinely reviewed and approved by the U.S. Food and Drug Administration (FDA) is hematopoietic stem cell transplantation to treat leukaemia and certain monogenic blood diseases [56].

Although still in the experimental stages, the concept of using stem cells to replace damaged neurons is now being explored in several neurodegenerative diseases, including ALS, where such therapies have shown some promise, albeit with transient effects. In ALS, cell replacement therapy using human foetal-derived neural stem cells has led to a transitory decrease in disease progression with no major deleterious effects in ALS patients [57]. Given that KAND is a monogenic disorder, in contrast to the complex genetics and environmental interactions that lead to ALS, cell replacement therapy for KAND could be potentially combined with ex vivo gene editing technology to supply patients with stem cells carrying a wild-type version of *KIF1A* to replace the neurons carrying variant *KIF1A*.

However, whilst potentially promising, there are significant challenges to overcome. These include ensuring efficient gene editing in stem cells, creating safe and effective protocols for stem cell transplantation, addressing potential immune responses and ensuring efficient integration of stem cell-derived neurons into the central nervous system. Whether these therapies would exert their effects by circuit integration or release of pro-survival factors is a major question that may underlie the transitory benefits seen in ALS. Nonetheless, with advances in gene editing and stem cell technologies, this approach holds considerable promise as a future treatment for KAND.

#### Gene therapy

Since KAND is a monogenic disorder, the use of gene therapy could be a promising option for treating KAND. Gene therapy using a range of delivery vectors and routes has been or is being tested in clinical trials for a number of neurodevelopmental disorders, with some examples summarised in Supplementary Table 1.

##### Gene replacement therapy

For loss-of-function and haploinsufficiency variants, gene replacement therapy could help by introducing additional functional wild-type KIF1A into the system. This increased expression could also counteract the effect of dominant-negative *KIF1A* variants by overwhelming the interfering impact that the mutant protein exerts on the wild-type protein’s function, thereby restoring overall cellular function.

KAND gene replacement therapy can be modelled using existing FDA-approved gene therapies, including Onasemnogene abeparvovec (Zolgensma®) for the treatment of Spinal Muscular Atrophy (SMA), caused by pathogenic variants in the *SMN1* gene, and voretigene neparvovecrzyl (Luxturna®) for the treatment of heritable retinal dystrophy caused by pathogenic variants in the *RPE65* gene. Both these therapies use adeno-associated virus (AAV) vector-based targeted delivery methods for their efficient delivery to the central nervous system or subretinal regions. These foundational successes underscore the feasibility of adapting similar AAV-based delivery methods for KAND gene replacement therapy, offering a promising avenue to address the underlying genetic defects and mitigate disease progression in multi-systems.

However, AAVs have their limitations, and one of these is their packaging capacity. The AAV vector has a packaging capacity of only 4.7 kilobases, which restricts its use to disorders whose coding sequence is less than this size limit. *KIF1A* coding sequence ranges from 1678 to 1816 amino acids (shortest experimentally determined protein isoform: NM_001379641.1, longest: NM_001379631.1), corresponding to 5034–5448 base pairs. One alternative approach would be to use dual AAV delivery for gene replacement therapy [58], which is currently being trialled for the human *HEXA* or *HEXB* genes for Tay-Sachs and Sandhoff diseases, respectively (ClinicalTrials.gov ID: NCT04669535). A second approach would be mini-gene-based gene replacement therapy, which is currently being trialled in Rett syndrome [59].

Alternatively, other viral and non-viral vectors could be employed for their potential to deliver genes to the central nervous system and large carrying capacity suitable for longer coding sequences. For instance, herpes simplex virus-1, with a potential capacity of 50 kb of foreign DNA, can travel via retrograde axonal transport from the infection site to the sensory neurons in the central nervous system, potentially making it an ideal tool for gene transfer therapy for neurological conditions [60].

##### Gene editing with CRISPR/Cas9

CRISPR/Cas9 technology offers a targeted approach to address gain-of-function variants in KAND by correcting or mitigating the effects of the pathogenic variants [61]. Precision editing methods, such as base editing and prime editing, can replace the mutant nucleotide sequence with the wild-type nucleotide, restoring normal gene function while minimising off-target effects. Alternatively, allele-specific strategies can selectively disrupt the mutant allele, leaving the wild-type allele unaffected, or use dCas9-based tools to silence mutant gene expression without permanent alterations. CRISPR-based gene regulation approaches, like CRISPRa/CRISPRi, could also balance the dominant-negative effects by modulating wild-type or mutant *KIF1A* expression. Currently, gene editing techniques using the CRISPR-Cas system have also been tested in clinical trials for *MECP2* duplication syndrome (ClinicalTrials.gov ID: NCT06615206) and have shown success in neurodegenerative disorders, genetic retinal diseases, hereditary hearing loss and immunodeficiency disease [62].

However, due to the heterogeneous variants underlying KAND, multiple gene editing systems may be required to correct different variants. In addition, challenges such as delivery to the central nervous system, potential off-target effects, immunogenicity, and ensuring cell-type specificity must be addressed to realise the full potential of CRISPR-based therapies for KAND. While gene-based therapies may offer the potential to slow or halt disease progression by targeting the underlying genetic cause in KAND, the benefits must be carefully balanced against significant uncertainties, including long-term safety, immune or inflammatory complications, genotoxicity risks, and the irreversibility of many interventions [63]. Regardless of whether individuals have a fast or slow progressive phenotype, it is critical to have a better understanding of the disease, captured by natural history studies, to determine whether gene therapies will be of clinical benefit.

## Concluding remarks

Continued advances in collaborative precision medicine research hold the key to unlocking transformative treatments and ultimately a cure for this devastating disorder.

This review highlights the promising potential of emerging therapeutic strategies for *KIF1A*-Associated Neurological Disorder (KAND), including gene replacement, gene editing, antisense oligonucleotides, and small molecule therapies. Advanced model systems, such as patient-derived iPSCs and animal models, have significantly enhanced our understanding of the pathophysiology of KAND and have accelerated drug discovery efforts. While challenges, including efficient delivery of targeted therapies and addressing KAND’s clinical diversity, remain, continued progress in precision medicine and collaborative research offers hope for developing transformative treatments and ultimately achieving a cure.

## Supplementary information

Selected gene therapies in neurodevelopmental disorders
